# Draft Genome Sequence of the Mycoparasitic Oomycete *Pythium oligandrum* Strain CBS 530.74

**DOI:** 10.1128/genomeA.00346-17

**Published:** 2017-05-25

**Authors:** Sandeep K. Kushwaha, Ramesh R. Vetukuri, Laura J. Grenville-Briggs

**Affiliations:** a Department of Plant Breeding, Swedish University of Agricultural Sciences, Alnarp, Sweden; b Department of Biology, NBIS, Lund University, Lund, Sweden; c Department of Plant Protection Biology, Swedish University of Agricultural Sciences, Alnarp, Sweden

## Abstract

The oomycete *Pythium oligandrum* is a mycoparasite and licenced biological control agent. Here, we report the draft genome sequence of *P. oligandrum* strain CBS 530.74, which is 36.80 Mb. It contains 341 scaffolds and 11,647 predicted protein-coding genes. As reported for plant-pathogenic *Pythium* species, RXLR-type effector sequences are absent.

## GENOME ANNOUNCEMENT

The oomycete *Pythium oligandrum* is an effective mycoparasite of economically important crop pathogens (1). It can directly parasitize the potato late blight pathogen *Phytophthora infestans* (2) and is amenable to genetic transformation (3). *P. oligandrum* establishes symbiotic relationships with plant roots actively promoting plant growth and also provides the plant with increased protection from other pathogens (1). Expression of elicitins activates plant defenses and thus primes the plant, protecting against subsequent attack (1). Mycoparasitism is relatively rare in the oomycete lineage, and most sequenced oomycetes are phytopathogenic. Thus, whole-genome sequencing of multiple strains of *P. oligandrum* will aid in the development of this species as a model for mycoparasitism. Insights from these analyses will facilitate sustainable control of plant diseases through a greater understanding of the mechanisms of both mycoparasitism and priming exhibited by this organism.

*P. oligandrum* was cultured and DNA extracted as described previously (4, 5). Paired-end (59.8 M reads) and mate-pair (78.5 M reads) libraries were sequenced using the Illumina HiSeqX sequencing platform (SciLifeLab, Sweden). FastQC and Trimmomatic tools were used for raw data quality assurance (QA) and quality control (QC), as described previously (5). The AllPaths software was used for *de novo* genome assembly (6). A total of 36.80 M bases were assembled into 341 scaffolds and all are more than 1 kb in length. QUAST (7) was used to assess assembly quality (*N*_50_, 645.019 bps; *N*_75_, 370,747 bps; *L*_50_, 21; *L*_75_, 40; longest scaffold, 1,427,593 bps; number of scaffolds >50 kb, 81; number of scaffolds >10 kb, 86). Assembly completeness was evaluated by BUSCO (8) based on a set of 1,438 common fungal genes as benchmark universal single-copy orthologs (BUSCOs); 797 complete genes and 213 fragmented and 408 missing BUSCOs have been found in this strain. The gene predictor Augustus (9) predicted 11,647 genes using the previously sequenced plant-pathogenic relative *Pythium ultimum* as a training model. Primary annotation of predicted sequences revealed 8,404 pfam domains, and SignalP analysis predicted 822 secretory proteins. Several predicted proteins have signatures matching Crinkler (CRN) effector proteins found in plant-pathogenic oomycetes. In contrast, effectors with signatures matching the largest class of secreted oomycete effectors (RXLR proteins) are apparently absent. An expansion of predicted CAZy proteins (10) compared to plant pathogens of the same genus was noted (E value, 1e-10), including glycoside hydrolases (342), glycosyltransferases (506), carbohydrate binding modules (480), polysaccharide lyases (14), carbohydrate esterases (93), and redox enzymes (18). Earlier Illumina Miseq sequencing of *P. oligandrum* Po37 assembled a draft genome from 14.51 M reads from paired sequencing (11). Our *P. oligandrum* CBS 530.74 assembly uses 138.3 M HiSeq reads (estimated 563× coverage) generated from two paired-end and two mate-pair libraries and thus provides complete and comparative data for genomic analysis of multiple strains of this species.

### Accession number(s).

This whole-genome shotgun project has been deposited at DDBJ/ENA/GenBank under the accession no. NAJK00000000. The version described in this paper is version NAJK01000000.
